# P-122. Evaluating the Clinical Impact of Metagenomic Next-Generation Sequencing in CNS Infections: Optimizing Diagnostic Pathways and Resource Utilization

**DOI:** 10.1093/ofid/ofaf695.349

**Published:** 2026-01-11

**Authors:** Gerome Vallejos, Amy Wong, Kiran T Thakur

**Affiliations:** New York Presbyterian Hospital / Columbia University Irving Medical Center, New York, New York; Delve Bio, Boston, Massachusetts; Columbia University Irving Medical Center, Orangeburg, NY

## Abstract

**Background:**

Diagnosing central nervous system (CNS) infections remains challenging due to the limitations of microbiological methods, which are often slow, target few pathogens, and often result in low diagnostic yield. Metagenomic next-generation sequencing (mNGS) offers a hypothesis-free, broad-range approach for pathogen detection, but its real-world impact on clinical decision-making is still being defined. This study aims to estimate the potential clinical utility of a mNGS test that detects pathogens in the cerebrospinal fluid (CSF) in ∼ 48h, using a Bayesian modeling framework.

**Methods:**

We utilized a cohort with confirmed CNS infections who underwent traditional, microbiological testing of CSF at CUIMC. Using clinical sensitivity and specificity data for mNGS, we applied Bayes’ theorem to calculate adjusted positive predictive values (PPVs) across different category-specific, pre-test probabilities. We then modeled the impact of implementing Delve Detect on the diagnostic workflow and cost savings, including the number of lumbar punctures (LPs), additional etiologic tests potentially avoided, and reduced days to diagnosis.

**Results:**

A total of 54 patients were divided into four category-specific etiologies: 23 DNA viruses, 5 RNA viruses, 16 bacterial, 7 fungus, and 3 parasitic. Using an mNGS test, such as Delve Detect could have reduced 88 microbiological tests, 145 days to diagnosis, and 2 LPs among 23 patients with DNA viral infections. For bacterial infections (n=16), it could have reduced 30 microbiological tests, 144 days to diagnosis and 12 LPs. Fungal infections (n=7) had an adjusted PPV of 92.8%, that could have potentially reduced 29 microbiological tests, 61 days to diagnosis and 3 LPs. Lastly, RNA viral and parasitic infections also showed benefit, with adjusted PPVs of 89.5% and 84.6% respectively, and modest reductions in testing and time to diagnosis.

**Conclusion:**

Our analysis suggests that mNGS could potentially streamline diagnostic and treatment pathways in CNS infections. By improving early diagnostic confidence, it may reduce the need for additional procedures and expedite targeted treatment. Given the reliance on modeling assumptions, future prospective studies are warranted to confirm these findings and support its integration into clinical practice.

**Disclosures:**

Amy Wong, PhD, Delve Bio: Employee Kiran T. Thakur, MD FAAN, Center for Disease Control: Grant/Research Support|Delve Bio: Advisor/Consultant|World Health Organization: Advisor/Consultant

